# High Prevalence of Sterile Pyuria in the Setting of Sexually Transmitted Infection in Women Presenting to an Emergency Department

**DOI:** 10.5811/westjem.2017.12.35605

**Published:** 2018-02-26

**Authors:** Stacia B. Shipman, Chelsea R. Risinger, Crystalle M. Evans, Chelsey D. Gilbertson, David E. Hogan

**Affiliations:** * Integris Southwest Medical Center, Department of Emergency Medicine, Oklahoma City, Oklahoma; † Norman Regional Health System, Department of Emergency Medicine, Norman, Oklahoma; ‡ Chickasaw Nation Medical Center, Department of Emergency Medicine, Ada, Oklahoma

## Abstract

**Introduction:**

The clinical presentations for sexually transmitted infections (STI) and urinary tract infections (UTI) often overlap, and symptoms of dysuria and urinary frequency/urgency occur with both STIs and UTIs. Abnormal urinalysis (UA) findings and pyuria are common in both UTIs and STIs, and confirmatory urine cultures are not available to emergency clinicians to aid in decision-making regarding prescribing antibiotics for UTIs. The objective of this study was to determine the frequency of sterile pyuria in women with confirmed STIs, as well as whether the absolute number of leukocytes on microscopy or nitrite on urine dipstick correlated with positive urine cultures in patients with confirmed STIs. We also sought to determine how many patients with STIs were inappropriately prescribed a UTI antibiotic.

**Methods:**

We performed a retrospective chart review of patients aged 18–50 who had a urinalysis and pelvic examination in the emergency department (including cervical cultures), and tested positive for *Neisseria gonorrhoeae, Chlamydia trachomatis,* and/or *Trichomonas vaginalis.* Descriptive statistics were obtained for all variables, and associations between various findings were sought using the Fisher’s exact test for categorical variables. We calculated comparison of proportions using the N-1 chi-squared analysis.

**Results:**

A total of 1,052 female patients tested positive for *Neisseria gonorrhoeae, Chlamydia trachomatis,* and/or *Trichomonas vaginalis* and were entered into the database. The prevalence of pyuria in all cases was 394/1,052, 37% (95% confidence interval [CI] [0.34–0.40]). Of the cases with pyuria, 293/394, 74% (95% CI [0.70–0.78]) had sterile pyuria with negative urine cultures. The prevalence of positive urine cultures in our study population was 101/1,052, 9.6% (95% CI [0.08–0.11]). Culture positive urines had a mean of 34 leukocytes per high-power field, and culture negative urines had a mean of 24 leukocytes per high-power field, with a difference of 10, (95% CI [3.46–16.15]), which was statistically significant (p=0.003). Only 123 cases tested positive for nitrite on the urinalysis dipstick; 50/123, 41% (95% CI [0.32–0.49]) had positive urine cultures, and 73/123, 59% (95% CI [0.51–0.68]) had negative urine cultures. Nitrite-positive urines were actually 18% more likely to be associated with negative urine cultures in the setting of positive STI cases, (95% CI [4.95–30.42], p=0.0048). Antibiotics were prescribed for 295 patients with suspected UTI. Of these, 195/295, 66% (95% CI [0.61–0.71]) had negative urine cultures, and 100/295, 34% (0.33, 95% CI [0.28–0.39]) had positive urine cultures. Chi-square analysis yielded a difference of these proportions of 32% (95% CI [23.92–39.62], p<0.0001).

**Conclusion:**

This study demonstrated that in female patients with STIs who have pyuria, there is a high prevalence of sterile pyuria. Our results suggest that reliance on pyuria or positive nitrite for the decision to add antimicrobial therapy empirically for a presumed urinary tract infection in cases in which an STI is confirmed or highly suspected is likely to result in substantial over-treatment.

## INTRODUCTION

Patients diagnosed with sexually transmitted infections (STI) are common in the emergency department (ED) setting. The Centers for Disease Control and Prevention (CDC) estimates that nearly 20 million new STIs occur annually.^1^ Patients undergoing evaluation for potential STIs will often have had comprehensive evaluation that includes gonococcal and chlamydia testing, wet prep, urinalysis, and urine culture. The clinical presentations for STIs and urinary tract infections (UTIs) may overlap, and symptoms of dysuria and urinary frequency/urgency occur with both STIs and UTIs.^2^,^3^,^4^ Abnormal urinalysis (UA) findings of leukocyte esterase and pyuria are common in both UTIs and STIs.^3^,^5^–^9^ STIs have been previously found to be associated with pyuria without bacteriuria.^2^,^10^–^11^ Furthermore, high STI rates have been reported in women evaluated in an urban ED and diagnosed with UTI.^12^–^14^

Emergency physicians (EP) must make decisions as to whether to empirically treat for UTIs based on initial UA results alone because confirmatory urine culture results are not readily available for several days after the patient’s ED visit. Findings of significant UA pyuria on these patients have the potential to lead EPs to treat the patient for a presumed “UTI” in patients who may actually have STIs and negative urine cultures.^15^,^16^ Additionally, nitrite-positive dipsticks have previously shown high specificity for UTIs,^17^–^19^ but this has not been studied specifically in STI-positive patients. Positive urine cultures have been defined by previous studies as growth of a bacterial pathogen >100,000 (10^5^) colonies.^6^,^10^ Sterile pyuria is classified as the presence of more than 5–8 leukocytes per high-power field on microscopy, in the setting of negative urine cultures.^4^,^20^–^21^

Treating a patient with sterile pyuria for a UTI can have negative effects, including antibiotic resistance and unnecessary cost to the patient.^7^ Antibiotic resistance and limited antibiotic selections are a worldwide public health concern. The patient taking an unnecessary antibiotic can have potential adverse effects, such as allergic reaction, anaphylaxis, or secondary, antibiotic-associated infection such as *C.difficile.*^22^ Antibiotic stewardship has become a responsibility for healthcare institutions and antibiotic prescribers, and recently a new standard of Joint Commission Requirements.^23^,^24^ The CDC identified that 20–50% of all antibiotics prescribed in U.S. acute care hospitals are either unnecessary or inappropriate.^23^ Not treating a UTI, on the other hand, can lead to pyelonephritis or even sepsis.^25^–^27^ This poses a dilemma for EPs trying to best treat these patients.

Previous studies in ED settings have demonstrated over-diagnosis of UTIs and under-diagnosis of STIs.^3^,^13^ However, prior studies have not specifically evaluated the incidence of sterile pyuria in patients with confirmed STIs. For EPs to provide their patients with optimal empiric antibiotic therapy, it can be helpful to identify whether patients with confirmed STIs commonly have associated culture-positive UTIs. The purpose of this study was to determine the frequency of sterile pyuria in patients with confirmed STIs (*Neisseria gonorrhoeae, Chlamydia trachomatis, and Trichomonas vaginalis*) seen in a community hospital ED. In addition, we examined the urine cultures of STI-positive patients who were prescribed an antibiotic for presumed UTI, and determined how many of those patients actually required antibiotics for positive urine cultures.

**Population Health Research Capsule**

What do we already know about this issue?

The clinical presentations for sexually transmitted infections (STI) and urinary tract infections (UTI) in females often overlap. Physicians may be empirically treating patients for UTIs based upon their initial urinalysis results, even if a STI is confirmed or strongly suspected.

What was the research question?

What is the prevalence of sterile pyuria in women with confirmed STIs?

What was the major finding of the study?

This study found an overall very low incidence of positive urine cultures in women with confirmed STIs, despite pyuria or positive nitrite on initial urinalysis.

How does this improve population health?

These findings have the potential to decrease unnecessary antibiotic prescriptions and overall improve antibiotic stewardship.

We hypothesized that STI-confirmed patients who have pyuria on initial urinalysis would have a high prevalence of sterile pyuria, as the urinalysis results were likely contaminated. We also hypothesized that prescribing UTI antibiotics for patients with suspected STI is unnecessary, and that the majority of these patients will have negative urine cultures.

## METHODS

### Study Design

We conducted a retrospective chart review of STI-positive, adult female patients who presented to the ED between January 2008 and December 2012. The chart abstractors were not blinded to the study hypothesis. The institution’s central institutional review board approved the study and granted exemption from informed consent.

### Study Setting and Population

All charts reviewed were from the ED at an urban, community, teaching hospital with over 85,000 patient visits annually and an associated emergency medicine residency program.

### Study Protocol

#### Inclusion Criteria

We included women in the retrospective chart review if they were age 18–50, had a urinalysis and pelvic examination in the ED (including cervical cultures), and tested positive for *Neisseria gonorrhoeae, Chlamydia trachomatis,* and/or *Trichomonas vaginalis.* Clinical judgment of the EP determined whether the patient had this initial work-up performed upon presentation.

### Data Collection

All endocervical cultures were obtained for gonorrhea and chlamydia testing using polymerase chain reaction (PCR) nucleic acid amplification and nucleic acid hybridization with the COBAS AMPLI-COR Analyzer (Roche, Indianapolis, IN). Samples of vaginal secretions were obtained for wet-mount preparation for detection of *Trichomonas* using a light microscope in the laboratory. Urinalysis was performed with the Clinitek ATLAS automated urine chemistry analyzer (Bayer Healthcare, Tarrytown, NY). A lab technician automatically performed microscopy of a centrifuged urine specimen, as well as urine cultures, if a greater than trace amount of protein, blood, nitrite, or leukocyte esterase was present. Urine cultures were plated with a 0.001-ml loop on MacConkey agars.

### Definitions

A positive urine culture was defined as growth of a known uropathogen ≥ 10^5^ CFU/ml. Pyuria was defined as more than five leukocytes per high-power field in a centrifuged urine sample.

### Outcome Measures

The primary outcome of the study was to determine the prevalence of sterile pyuria in patients with confirmed STIs. Secondary outcomes included the rate of positive urine cultures in women who tested nitrite positive in the study population. Additionally, we sought to determine the number of patients treated with antibiotics for suspected UTI who had negative urine cultures.

### Data Analysis

We entered data without patient identifiers into a custom database constructed in Microsoft Excel (version 14.0.7140.5002. ©Microsoft Corp. 2010) and performed analysis with the statistical add-on package Analyze-it, version 2.26 Excel 12+. We sought associations between various findings using Fisher’s exact test for categorical variables. Comparison of proportions was calculated using the N-1 chi-squared analysis. We set significance at p<0.05 throughout.

## RESULTS

During the study period, we entered 1,052 cases into the database. All cases were female patients who tested positive for *Neisseria gonorrhoeae, Chlamydia trachomatis,* and/or *Trichomonas vaginalis.* The mean age was 22.9 years with a range of 14 to 51. The prevalence of each disease in the dataset were the following: gonorrhea 351/1,052, 33% (95% confidence interval [CI] [0.30–0.36]) chlamydia 853/1,052, 81% (95% CI [0.79–0.83]); trichomonas 176/1,052, 17% (95% CI [0.14–0.19]).

The prevalence of pyuria in all cases entered into the database was 394/1,052, 37% (95% CI [0.34–0.40]). Of the cases with pyuria, 293/394, 74% (95% CI [0.70–0.78]) had sterile pyuria with negative urine cultures. The prevalence of positive urine cultures in our total study population was 101/1,052, 9.6% (95% CI [0.08–0.11]) (Figure). Further review of the initial urine-microscopy results of STI-positive patients with pyuria showed that both culture-positive and culture-negative urines had a range of 6–100 leukocytes per high-power field. Culture-positive urines had a mean of 34 leukocytes per high-power field, and culture-negative urines had a mean of 24 leukocytes per high-power field, with a difference of 10, (95% CI [3.46–16.15]), which was statistically significant (p=0.003).

We additionally reviewed the data to examine if nitrite in the urinalysis of these STI-positive cases correlated with positive culture results. Only 123 cases tested positive for nitrite on the urinalysis dipstick; 50/123, 41% (95% CI [0.32–0.49]) had positive urine cultures, and 73/123, 59% (95% CI [0.51–0.68]) had negative urine cultures. Nitrite-positive urines were actually 18% more likely to be associated with negative urine cultures in the setting of positive STI cases (95% CI [4.95–30.42], p=0.0048).

In our retrospective review of the 1,052 cases, 295 patients were prescribed antibiotics for suspected UTI. These antibiotics included cephalexin (206), ciprofloxacin (50), nitrofurantoin (36), sulfamethoxazole/trimethoprim (2), and amoxicillin (1). Of these, 195/295, 66% (95% CI [0.61–0.71]) had negative urine cultures, and 100/295, 34% (0.33, 95% CI [0.28–0.39]) had positive urine cultures. Chi-square analysis yielded a difference of these proportions of 32% (95% CI [23.92–39].62, p<0.0001). Of those 100 patients who had positive urine cultures, six grew a pathogen resistant to the antibiotic given for UTI.

## DISCUSSION

Previous studies have found that women with urinary symptoms are over-diagnosed with UTI and under-diagnosed with STIs,^3^,^13^,^28^ but no prior research has specifically analyzed urine results of known STI-positive patients. In this retrospective review of women testing positive for *Neisseria gonorrhoeae, Chlamydia trachomatis,* and/or *Trichomonas vaginalis* over a five-year period at a large metropolitan ED, we found that of the cases with pyuria, 74% of those were sterile pyuria. Our study found a very low overall incidence of positive urine cultures (9.6%) in the setting of women with positive STIs. Of the patients with pyuria, patients with culture-positive urines vs. culture-negative urines had identical ranges of urine leukocytes (6–100 leukocytes per high-power field), but the mean leukocytes were higher in the culture-positive group (33.842 versus 24.034 leukocytes per high-power field).

Prior literature indicates that in the general population the urine-dipstick, nitrite reaction has a low sensitivity but a very high specificity, making a positive result useful in confirming the diagnosis of UTI caused by organisms capable of converting nitrates to nitrite such as *Escherichia coli*.^15^,^17^,^21^ However, the urine-dipstick test for nitrites has not been studied in STI-positive patients. We found that in the setting of positive STI cases, positive nitrite on the urine dipstick is not a good indication of UTI. Our results showed that in STI-positive cases, nitrite-positive urines were actually 18% more likely to be associated with negative urine cultures.

Current scientific literature emphasizes the need to reduce the use of inappropriate antimicrobials in all healthcare settings due primarily to antimicrobial resistance, but also because of the associated costs and potential adverse effects (including allergic reactions and development of secondary antibiotic-associated infections such as *C.difficile*).^22^–^24^, ^29^,^30^ Our study found that of the 295 patients with confirmed STIs who were also prescribed an antibiotic for a presumed UTI, 66% of those were unnecessary, as they had negative urine cultures.

## LIMITATIONS

The primary limitations of this study were its retrospective in nature and that it was performed at a single center; however, we obtained sufficient numbers of cases with full datasets to keep the data quality robust. All of the cases in the study were also positive for an STI, as it was retrospective, and all of their culture results were confirmed. This limits the EP to generally apply the results to a specific population (i.e., women who present with dysuria or pelvic pain), as they may not know the patient has a STI at the time of the visit. Another limitation is that we defined a UTI using the previously defined “microbiologic definition” of >100,000 colony-forming units.^6^,^10^,^31^ Some other studies have defined a UTI with “low-count” colony criteria of 10^2^–10^3^ CFU/mL;^3^,^28^ had we used a lower threshold we might have calculated more “culture-positive” urines. Additionally, the chart abstractors were not blinded to the study hypothesis, which could have introduced potential bias.

## CONCLUSION

This study demonstrates that in female patients with STIs who have pyuria, there is a high prevalence of sterile pyuria. Our results suggest that reliance on pyuria or positive nitrite for the decision to add antimicrobial therapy empirically for a presumed UTI in cases in which an STI is confirmed or highly suspected is likely to result in substantial over-treatment.

## Figures and Tables

**Figure:**
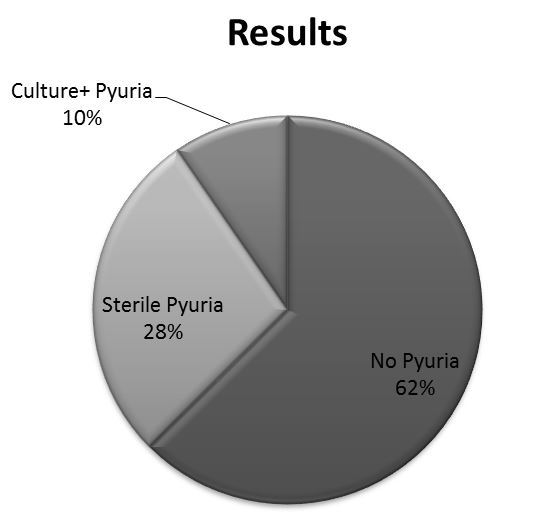
Prevalence of pyuria in female patients with documented sexually transmitted infections.
